# Virulence evolution of a generalist plant virus in a heterogeneous host system

**DOI:** 10.1111/eva.12073

**Published:** 2013-05-20

**Authors:** Mónica Betancourt, Fernando Escriu, Aurora Fraile, Fernando García-Arenal

**Affiliations:** 1 Centro de Biotecnología y Genómica de Plantas UPM-INIA and E.T.S.I. Agrónomos, Universidad Politécnica de Madrid, Campus de MontegancedoMadrid, Spain; 2 Centro de Investigación y Tecnología Agroalimentaria de Aragón, Unidad de Sanidad VegetalZaragoza, Spain

**Keywords:** *Cucumber mosaic virus*, multihost parasites, virulence evolution, virus emergence

## Abstract

Modelling virulence evolution of multihost parasites in heterogeneous host systems requires knowledge of the parasite biology over its various hosts. We modelled the evolution of virulence of a generalist plant virus, *Cucumber mosaic virus* (CMV) over two hosts, in which CMV genotypes differ for within-host multiplication and virulence. According to knowledge on CMV biology over different hosts, the model allows for inoculum flows between hosts and for host co-infection by competing virus genotypes, competition affecting transmission rates to new hosts. Parameters of within-host multiplication, within-host competition, virulence and transmission were determined experimentally for different CMV genotypes in each host. Emergence of highly virulent genotypes was predicted to occur as mixed infections, favoured by high vector densities. For most simulated conditions, evolution to high virulence in the more competent Host 1 was little dependent on inoculum flow from Host 2, while in Host 2, it depended on transmission from Host 1. Virulence evolution bifurcated in each host at low, but not at high, vector densities. There was no evidence of between-host trade-offs in CMV life-history traits, at odds with most theoretical assumptions. Predictions agreed with field observations and are relevant for designing control strategies for multihost plant viruses.

## Introduction

A major topic of evolutionary biology is the study of infectious diseases, and the evolution of virulence, defined as the negative effect of infection on host fitness (Read 1994), has been extensively modelled. Models assume trade-offs between parasite life-history traits, mostly between transmission and virulence, by considering different factors and, have identified the selective forces acting on parasites (Bull 1994; Frank 1996; Lipsitch and Moxon 1997; Ebert and Bull 2003; Gandon and Day 2003; Day and Proulx 2004; Alizon et al. 2009). Most work has focused on single-host, obligate parasites (Gandon 2004; Brown et al. 2012; Williams 2012). This is in spite that a large fraction of pathogens of humans, other animals and plants are generalists or multihost parasites, that is, they are able to infect different hosts belonging to different taxa (Woolhouse et al. 2001), and that generalists may be, or behave as, opportunists for a focal host (Woolhouse et al. 2001; Haydon et al. 2002; Brown et al. 2012). Analyses considering multiple hosts identify among-host heterogeneity in resistance and virulence, costs of infecting different hosts and differences in within-host and between-host transmission rates, as major factors driving the evolution of generalist parasites, and mostly predict that virulence will evolve to levels below the optima for each host (Ebert and Hamilton 1996; Regoes et al. 2000; Gandon et al. 2002; Dobson 2004; Gandon 2004; Williams 2012; but see Ganusov et al. 2002). As is the case for single-host analyses, there is a general paucity of experimental data on the values of key parameters in models, and empirical tests of theoretical predictions have not been frequent (Pfennig 2001; Gandon 2004; van den Bosch et al. 2006; Jeger et al. 2006). This is particularly so for plant pathogens, for which even the basic assumption of a trade-off between parasite virulence and transmission has been evaluated in few instances (Jarosz and Davelos 1995; Sacristán and García-Arenal 2008).

The purpose of this work is to analyse the factors that drive the evolution of virulence of a generalist plant virus, *Cucumber mosaic virus* (CMV, family *Bromoviridae*). CMV has a single-stranded, messenger-sense RNA genome built of three segments that are separately encapsidated in isometric particles. CMV has the broadest host range described for a plant virus, infecting more than 1200 species in more than 100 plant families. CMV is transmitted by more than 80 species of aphids (Hemiptera: Aphididae). Transmission is nonpersistent, that is, the virus does not infect the insect vector, but is retained in its mouth parts, and the aphid is able to transmit the virus for a short time (<2 h) after acquisition from an infected plant. CMV is also transmitted through the seed, with efficiency varying largely according to the plant species. Seed transmission may be epidemiologically relevant in weed reservoirs that, together with other crops, are inoculum sources for epidemics in crops (for a review on CMV, see Jacquemond 2012). CMV is the helper virus for a satellite RNA (satRNA), which is a small, noncoding, single-stranded RNA, not infectious by itself but depends on CMV for its replication, encapsidation and transmission. The presence of a satRNA results in a depression of CMV accumulation in the infected plant, so that it behaves as a molecular parasite of CMV. CMV-satRNA may modulate the pathogenicity of CMV in a way that depends on the strains of CMV and satRNA and on the species of host plant. While most satRNA variants do not modify or attenuate CMV symptoms in most plant species, in tomato, two main phenotypes can be distinguished, those that attenuate CMV symptoms (A-satRNAs) and those that aggravate them to a systemic necrosis (N-satRNAs). Most described CMV isolates do not support a satRNA, and CMV-satRNAs occur with low frequency in the field; high satRNA prevalence has been mostly associated with epidemics of tomato necrosis (for reviews on CMV-satRNA, see García-Arenal and Palukaitis 1999; Palukaitis and García-Arenal 2003).

From 1986 to 1992, one such epidemics of systemic necrosis occurred in tomato crops in eastern Spain, caused by CMV plus satRNAs (Jordá et al. 1992; Escriu et al. 2000a). CMV isolates collected during this epidemic caused three different symptoms in tomato plants: a systemic necrosis (N isolates), a stunting of the plant and curling of the leaves (A isolates) and a stunting of the plant with extreme reduction in the leaf lamina (Y isolates). N and A isolates were associated with satRNA-variants necrogenic and non-necrogenic (i.e. attenuative of CMV symptoms), respectively, while Y isolates were not associated with satRNAs (Jordá et al. 1992). The symptoms caused by N and A isolates were determined solely by the presence and nature of the associated satRNA and not by the interaction between satRNA variant and CMV variant (Escriu et al. 2000a). It should be noted that satRNAs associated with CMV isolates during this epidemic showed high genetic variation due to mutation accumulation and recombination but had only two phenotypes on tomato plants, necrogenic and attenuative as described above; attenuative and necrogenic satRNAs belonged to two clearly different evolutionary lineages (Aranda et al. 1993, 1997; Escriu et al. 2000a). In other host species, isolates Y, N and A did not obviously differ in symptoms, but a deeper analysis showed that in melon plants, in spite that Y, N and A isolates all caused a similar leaf mosaic, A and N isolates reduced plant growth similarly and more severely than Y isolates (Betancourt et al. 2011). Thus, CMV virulence in different host plant species is genetically determined, as it is modulated by the presence of satRNAs that can be considered as a fourth nonessential component of the genome of CMV.

Some years ago, we analysed the factors leading to the emergence of the tomato necrosis syndrome, that is, the factors that determined the invasion of the CMV population by N isolates. For this, model parameters for within-host multiplication, competition in mixed infections, virulence and transmission were determined experimentally for N, A and Y isolates (Escriu et al. 2000a,b). A model that allowed co-infection of a single host by different isolate types, and competition between types with an effect on transmission explained satisfactorily the invasion of the CMV population by N isolates at the beginning of the tomato necrosis epidemic, and its predictions also agreed with the long-term evolution of the CMV population according to field data. Important conclusions from this analysis were that the invasion of the CMV population by N isolates occurred in co-infection with A isolates and required high densities of the aphid vector's population (Escriu et al. 2003). In that analysis, the role that other CMV hosts in which N and A isolates would not have a specific phenotype (i.e. the large majority of CMV hosts) could play in N isolates emergence was not considered. This is the goal of the present work.

Here, we extend the analysis of CMV virulence evolution to a system considering two host species, among which isolates of N, A and Y genotypes will differ in within-host multiplication, competition in mixed infections, virulence and transmission. In addition to tomato, the focal host in which the necrosis epidemic emerged, melon was chosen as the second host. As satRNA variants responsible for the N and A CMV types in tomato do not differ in phenotype in melon plants (Betancourt et al. 2011), melon can be considered as representative of the large majority of CMV host plant species in this respect. Also, melon shares with most vegetables and weeds the trait of being a poorer host of CMV-satRNA than tomato and other species from the *Solanaceae* (García-Arenal and Palukaitis 1999; Betancourt et al. 2011). Last, melon is the most important CMV host crop sharing a geographical area, and overlapping in time, with tomato crops in Mediterranean Spain, where the epidemic of tomato necrosis occurred. Results indicate that the rate of transmission, determined by the density of the aphid vector population, is the key factor in CMV virulence evolution. Results also show that between-host and within-host transmission rate variation determines the possibility of emergence of highly virulent isolates in either hosts, but has different effects on the dynamics of CMV infection in each host.

## Models

### Models description

We have used SIR-like models allowing for co-infection of a single host, with within-host competition among co-infecting isolates, which will influence transmission rates. These models were derived from that initially proposed by Mosquera and Adler (1998). An important difference is that recovery of infected plants is not considered, as CMV causes systemic persistent infections so that plants, once CMV-infected, remain so until the end of their life cycle. We used epidemiological models in which mutations having an effect in virulence were not considered, as our previous results indicated that conversion of A-satRNAs into N-satRNAs, or *vice versa*, by mutation or recombination would be extremely rare events (Aranda et al. 1997; Escriu et al. 2000a). For a single host, the dynamics of the model is described by the equations (Escriu et al. 2003):


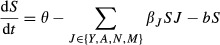
(1a)


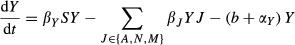
(1b)


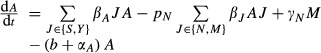
(1c)


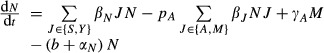
(1d)


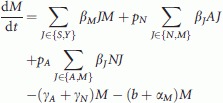
(1e)

Equations represent the variation with time (days) of density (plants/m^2^) of susceptible noninfected plants (*S*) or plants infected by isolate *J* (*J* being isolates Y, A, N and M, M indicating mixed infection by A and N isolates, *J* in capitals for populations of infected plants or as subscripts for model parameters). Parameter *α*_*J*_ indicates the virulence of isolate *J*, expressed as the increase in *per capita* host mortality rate due to infection. The transmission rate *β*_*J*_ represents the number of virus transmissions per unit time per infected host per available susceptible host. CMV isolates Y, A and N can infect healthy susceptible plants (*S*) resulting in *Y-, A-* and *N-*infected plants. Besides, A and N isolates can infect *Y* plants, resulting in *A* and *N* plants, because acquisition of satRNAs from A or N isolates by Y isolates will convert these into A and N isolates, respectively. Last, A or N isolates can infect *N* or *A* plants, respectively, resulting in a new population of A+N mixed-infected plants (*M*). Parameter *p*_*J*_ (*J* = A, N) represents the frequency of success of parasite *J* in competing with an established parasite (i.e. N, A) when infecting an already infected plant, resulting in flows into the *M* plant class. *γ*_*A*_ and *γ*_*N*_ represent the rate per plant and unit time at which A isolates are displaced by N isolates, or *vice versa*, respectively, when A and N isolates compete within *M* plants, resulting in flows from *M* class plants. Parameters *p*_*J*_ and *γ*_*J*_ are related through the Lotka–Volterra competition model, as further explained below (*Estimation of competition parameters*). Note that the flow from *A, N* or *M* to *Y* plants is not considered. This simplification was introduced as it was experimentally shown that the fraction of transmissions from *N* or *A* plants resulting in *Y* plants was negligible in tomato and much lower than the fraction resulting in *N* or *A* plants in melon (Escriu et al. 2000b; Betancourt et al. 2011). Also, it was considered that transmission of A+N isolates from *M* plants is much more probable than that of A or N isolates alone: if the proportion of isolates A and N in *M* plants is *f*_*A*_ and *f*_*N*_, and a transmission event involves *k* virus particles, A and N isolates will be transmitted with probabilities *f*_*A*_^*k*^ and *f*_*N*_^*k*^, and the probability of transmission of A+N isolates will be 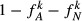. Note that during nonpersistent aphid transmission, it is assumed that virus particles are sampled at random from the source virus population (Betancourt et al. 2008). Although the effective number of particles transmitted by a single aphid is small (Betancourt et al. 2008), as soon as more than one aphid is involved in a transmission event 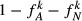 will be much bigger than 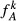 and 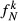.

We considered a monomolecular growth of the population of susceptible plants. For any host *H*, *θ*_*H*_ = *r* (*K*_*H*_−*T*), where *T* is the total plant population, *K* is the maximum size of the population and *r* is its rate of growth. In both crops, plant populations stay constant during a growing season, so we set *r* = 1 to get a constant value of *T* = *K*, that is, the crop does not change with time. According with the crop conditions, *K* was of 4 plants/m^2^ for tomato (Host 1) and of 0.8 plants/m^2^ for melon (Host 2). Parameters are described in Table 1, and a full description of this model is in Escriu et al.'s study (2003).

**Table 1:** Estimates of parameters of virulence, transmission and competition for *Cucumber mosaic virus* (CMV) genotypes in two hosts, tomato and melon

Parameters	Description	Tomato* (Host 1)	Melon† (Host 2)
*b*	*Per capita* mortality rate of uninfected plants	0.00952	0.01000
*α*_*Y*_	*Per capita* plant mortality rate increase due to infection by isolates Y	0.00142	0.00150
*α*_*A*_	*Per capita* plant mortality rate increase due to infection by isolates A	0.00004	0.01413
*α*_*N*_	*Per capita* plant mortality rate increase due to infection by isolates N	0.01120	0.01311
*α*_*M*_	*Per capita* plant mortality rate increase due to infection by isolates M (= A+N)	0.01120	0.01220
*β*_*pY*_ (*i* = 1)	Probability of transmission of isolates Y for each aphid-mediated contact	0.52795	0.32703
*β*_*pA*_ (*i* = 1)	Probability of transmission of isolates A for each aphid-mediated contact	0.28771	0.25088
*β*_*pN*_ (*i* = 1)	Probability of transmission of isolates N for each aphid-mediated contact	0.19979	0.17965
*β*_*pM*_ (*i* = 1)	Probability of transmission of isolates M (= A+N) for each aphid-mediated contact	0.19979	0.17965
*p*_*A*_	Frequency of success of isolates A at infecting a plant already infected by isolates N	0.83	1
*p*_*N*_	Frequency of success of isolates N at infecting a plant already infected by isolates A	1	1
*γ*_*A*_	*Per capita* rate at which isolates A are displaced by isolates N through within-plant competition	0.0113	0
*γ*_*N*_	*Per capita* rate at which isolates N are displaced by isolates A through within-plant competition	0	0

* Data from Escriu et al. 2003.

† Derived from data in Betancourt et al. 2011 as explained in main text.

This model was extended to two hosts, and its dynamics for Host 1 are described by the set of equations:


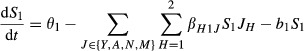
(2a)


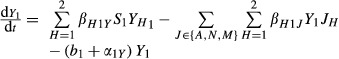
(2b)


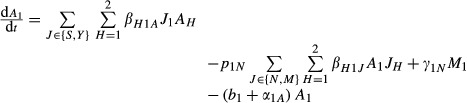
(2c)


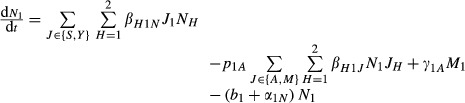
(2d)


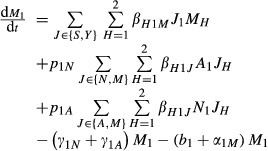
(2e)

Subscripts 1 and 2 denote the host, and the model differs from the single host one (Eqn. 1) in that it allows for infection of Host 1 from Host 2 (parameters *β*_21*J*_) in addition to the infection of Host 1 from Host 1 (parameters *β*_11*J*_). A second difference is the parameter *H*, denoting the host plant species that may be *H* = 1 for Host 1 and *H* = 2 for Host 2. The flow diagram for the model is shown in Figure S1. A similar set of equations describes the dynamics for Host 2 (not shown).

### Estimation of model parameters

The values of the parameters in the models above had been estimated experimentally for tomato, and the experimental procedures and values have been reported previously (Escriu et al. 2003). The same methodology was used for the estimation of parameters for melon, based on previously published results on the interaction of CMV and satRNAs with this host plant (Betancourt et al. 2011). Melon plants (*Cucumis melo* L) cv. Piel de Sapo were used in all experiments. As is the case for all melon cultivars grown in Spain, Piel de Sapo is fully susceptible to CMV. For all experiments, CMV strain Fny (Fny-CMV, Owen and Palukaitis 1988) was used alone (Y isolate) or as a helper virus for ten satRNA genetic variants with a necrogenic phenotype in tomato (N isolates) and ten satRNA genetic variants with a non-necrogenic (i.e. attenuative) phenotype in tomato (A isolates). These satRNAs were randomly chosen from a collection of satRNA isolates from the field and were the same used previously to estimate model parameters for tomato (Escriu et al. 2000a). Both CMV and satRNAs were derived from infectious RNA transcripts of full-length cDNA clones (Rizzo and Palukaitis 1990; Escriu et al. 2000a) to minimize mutation accumulation and selection during experimentation in different host plants.

#### Estimation of virulence

As is the case for most plant viruses, CMV infection is not lethal, with the exception of N isolates in tomato. Hence, it is difficult to quantify virulence as the instantaneous mortality rate and, following Day (2002), virulence was quantified as the reduction in the host expected lifespan by infection. Instantaneous mortality rates relate to lifespan by *b* = 1/*Ds* for noninfected plants or by (*b* + *α*_*J*_) = 1/*D*_*J*_ for plants infected by isolate *J*, being D_S_ and D_*J*_ the lifespan of healthy and *J*-infected melon plants, respectively. *D*_*s*_ was estimated as of 100 days according to the agricultural practices in Spain (Alonso-Prados et al. 2003), hence, *b* = 0.01/day. *D*_*J*_ was estimated experimentally in the form *D*_*J*_ = *d*_*J*_ · *D*_*S*_, where *d*_*J*_ represents the survival of *J*-infected plants relative to healthy ones.

As reported for tomato (Escriu et al. 2003), both for infected and mock-inoculated melon plants, a linear regression was found between the lifespan of each leaf (leaf survival, *LS*) and the square root of its biomass plus that of all previously senesced leaves of the same plant (senescent biomass, *SB*)*,* that is, the lifespan of each leaf was dependent on the previous growth of the plant. The slope of the linear regression of *LS* on the square root of *SB* was significantly different between mock-inoculated and CMV-infected plants, but did not differ among Y-, N- and A-infected plants (Betancourt et al. 2011). The infection of melon plants by CMV had the effect of significantly reducing plant growth as compared to mock-inoculated controls. Growth was more severely reduced by N and A isolates than by Y isolates, but there were no significant differences between the growth of plants infected by N and A isolates or by A+N isolates in mixed infections (Betancourt et al. 2011). With these data, values for *d*_*J*_ and *α*_*J*_ were calculated for each Y, N and A isolate and for mixed infections between N and A isolates (Table S1). Mean values of *α*_*J*_ for each type of isolate are shown in Table 1, showing that N and A isolates were similarly virulent on melon and were more virulent than Y isolates; virulence in mixed infections did not differ significantly from virulence of A and N isolates in single infection. Because differences in plant growth or virulence between N, A and M isolates were nonsignificant, mean virulence values could have been used in simulations, as well as those in Table 1. The use of mean virulence values did not affect any of the reported results (not shown). Hence, virulence of the different types of CMV isolates was not the same for both hosts, because for tomato, N isolates were the most virulent, Y isolates had an intermediate virulence, and A isolates had very low virulence; virulence of mixed infections was as that of N isolates (Table 1).

#### Estimation of transmission rates

The transmission rate for each CMV isolate, *β*_*J*_*,* was considered as the product of two terms *β*_*J*_ *= β*_*e*_*(i) · β*_*pJ*_*(i) a*s in Escriu et al.'s study (2003). The first term represents the number of aphid-mediated contacts between plants, that is, the number of events per unit time and plant in which one (or several) aphid(s) leaves an infected plant and feeds in another one; the second term is the probability of virus transmission of isolate *J* for each of these events (Day 2001). Both *β*_*e*_ and *β*_*pJ*_ may vary with the number of aphids per plant, *i*.

In both melon and tomato, the frequency of transmission by *Aphis gossypii* for one single aphid (*i* = 1) was shown to be determined by virus accumulation levels in the source leaf, although the relationship between both variables differed for each host (Escriu et al. 2000b; Betancourt et al. 2011). Similarly to tomato, accumulation levels of Y, N and A isolates differed significantly in melon plants, being smaller for N isolates, intermediate for A isolates and highest for Y isolates (Betancourt et al. 2011). With these data, values of the probability of transmission of each isolate by a single aphid *β*_*pJ*_ (*i* = 1) were calculated and are shown in Table S2, and average values for each type of isolate are shown in Table 1. Note that *β*_*pJ*_ (*i* = 1) values ranged similarly for the different types of isolates in both hosts (i.e. Y>A>N), but because CMV multiplication is more efficient in tomato than in melon (see below), absolute values are higher in this host (Table 1) (Escriu et al. 2000a; Betancourt et al. 2011). As values of *β*_*pJ*_ (*i* = 1) depended on the virus accumulation levels in the source leaf, for between-host transmissions, we assumed that *β*_*pJ*_ from melon to tomato was as *β*_*pJ*_ for melon and that *β*_*pJ*_ from tomato to melon was as *β*_*pJ*_ for tomato.

*β*_*pJ*_*(i)* was calculated for other *i* values according to the expression proposed by Gibbs and Gower (1960): *β*_*pJ*_*(i) =* 1− [1−*β*_*pJ*_ (*i =* 1*)*]^i^. Note that as more aphids participate in each transmission event, that is, the higher the *i* value, the smaller the difference in *β*_*p*_*(i)* values for the three CMV genotypes (Figure S2). We were unable to estimate experimentally the rate of transmission events *β*_*e*_ for any value of *i* and have used arbitrary values of *β*_*e*_*(i)* varying between 0.0001 and 0.1/days; these values may be realistic as epidemiological studies of CMV in different regions of Spain for different years indicate transmission rates of 0.008–0.122/days (Alonso-Prados et al. 2003).

#### Estimation of competition parameters

Parameters *p*_*A*_*, p*_*N*_*, γ*_*A*_ and *γ*_*N*_ depend on competition between A and N isolates in *M* plants. Dynamics of competition was simulated by the logistic equations of Lotka–Volterra model (Bulmer 1994). Our previous results had shown that in melon plants, the accumulation in single infection of N-satRNAs was more efficient than the accumulation of A-satRNA; indeed, four of ten assayed A-satRNA did not accumulate to detectable levels in systemically infected melon leaves (Betancourt et al. 2011; see also Tables S1 and S2). In mixed infections, the accumulation of N-satRNAs was significantly depressed as compared to single infections (0.35 ± 0.01 μg satRNA per g of leaf in mixed vs 0.46 ± 0.03 μg/g in single infections), while accumulation of A-satRNAs was unaffected by the presence of N-satRNAs (0.16 ± 0.02 μg/g in mixed vs 0.14 ± 0.01 μg/g in single infection; Table 1 in Betancourt et al. 2011). These data were used to estimate the competition parameters *c*_*ij*_ (inhibitory effect of parasite *j* on parasite *i*) in the model of Lotka–Volterra, giving the values *c*_AN_ = 0 and *c*_NA_ = 0.733. These values were used in 100 simulations of the competition model, letting them vary at 10% (close to the standard error of the original data on accumulation). The resulting competition dynamics for A and N isolates in *M* plants was given as follows: frequency of co-existence of genotypes A and N when infecting a plant already infected by the N or A genotype, *p*_*A*_ = *p*_*N*_ = 1, and frequency of displacement of A by N and of N by A *γ*_*A*_ = *γ*_*N*_ = 0 (Table 1). Thus, although A isolates inhibited N-isolate multiplication in mixed infections, this effect was not so strong than N isolates were displaced; Lotka–Volterra frequencies at equilibrium being 0.301 ± 0.0004 and 0.699 ± 0.0004 for A and N isolates, respectively, in good agreement with experimentally determined values.

In summary, the behaviour of N and A types in both hosts is broadly different: in tomato, N- and A-satRNAs accumulated to similar levels in single infection, but N-satRNAs successfully outcompeted A-satRNA in *M* plants. In melon, N-satRNAs accumulated to higher levels than A-satRNA in single infection, but suffered the effect of competition of A-satRNAs in mixed infection (Table 1). Note also that the multiplication of any type of sat RNA in melon was much lower than in tomato, about 75-fold lower for N-satRNAs and about 200-fold lower for A-satRNAs (Escriu et al. 2000a; Betancourt et al. 2011).

## Results

### Evolution of CMV virulence in the melon crop

**Figure 1:**
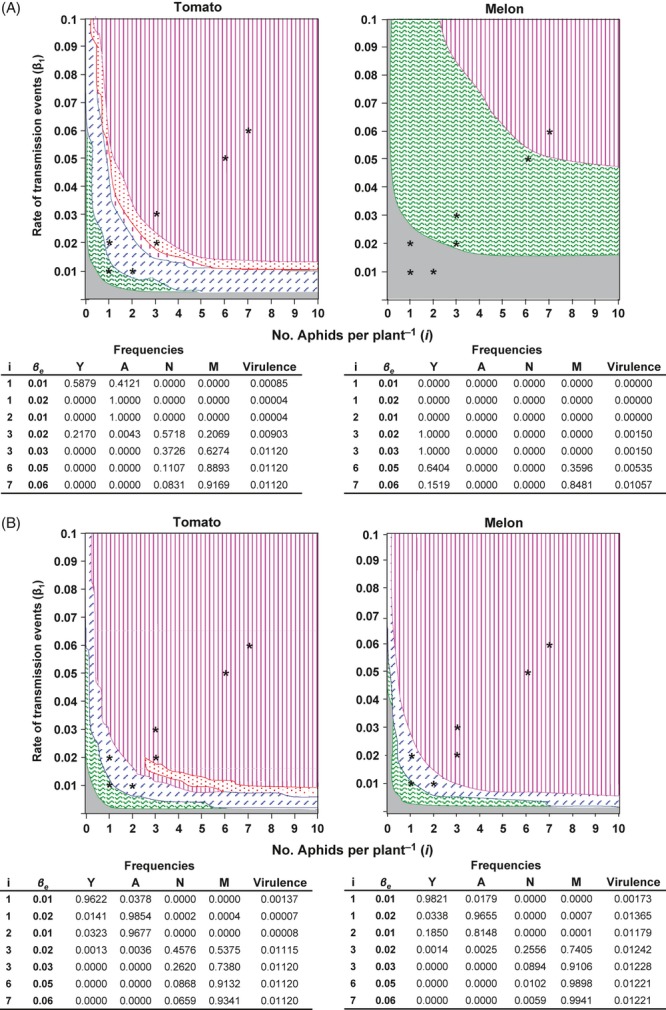
Predictions of co-infection models for one host (A) or for two hosts (B) for virulence evolution of CMV in Host 1 (tomato) and Host 2 (melon), as a function of the number of aphids per plant (*i*) and of the rate of transmission events (*β*_*e*_). Graphs indicate areas in which there is no infection (S 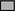 ) or where Y isolates (Y 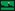 ), A isolates (A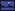 ), N isolates (N 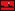 ) or A+N isolates (M 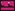 ) are the most prevalent in the virus populations. Figure 1A for tomato was redrawn from the study by Escriu et al. (2003). For a series of *i* and *β*_*e*_ values, indicated by asterisks in the figure, the relative equilibrium frequency of the different virus genotypes in, and the average virulence of, the virus population is indicated.

We analysed first the evolution of CMV virulence in the melon crop by itself. Isolates *J* (*J* = Y, N and A) differed in their basic reproductive value, *R*_0_ = *β*_*J*_*T*/(*b*+*α*_*J*_) (Frank 1996). Both in tomato and melon, *R*_0_ values ranked Y>A>N at low or moderate aphid densities. As the aphid density, *i*, increased, *R*_0_ values increased and differences between Y, N and A isolates decreased, so that at high *i* values, *R*_0_ for Y and A isolates in tomato, and for A and N isolates in melon, did not differ (Table 2). Maximization of *R*_0_, however, did not explain the invasion of the CMV population by N isolates. As previously shown for tomato, the invasion of the CMV population by N isolates was only predicted using the co-infection model represented by eqn (1). Simulations of this model were done for *β*_*e*_ values between 0.01 and 0.15 and for *i* values of 0.5–30 aphids per plant, and with initial conditions of *S* = 0.77, *Y = N = A* = 0.01, *M* = 0 plants/m^2^. These simulations yielded data on the density of plants infected by isolates Y, A, N and M (*Y, A, N* and *M* plants), on which the relative frequency of Y, A, N and M isolates in the virus population could be determined and hence the average virulence of the population. Relative isolate frequencies and average virulence varied with time (i.e. evolved) until reaching an equilibrium that differed under different scenarios (Fig. 1A). The model predicted that invasion of the CMV population by N or A isolates occurred mostly in mixed infections (*M* plants). Also, as previously shown for tomato, the major factor determining the invasion of the CMV population by N isolates was the density of the aphid population. For a rate of transmission events *β*_*e*_ = 0.03, Y-CMV isolates become the most prevalent in the melon population when the density of aphids exceeded 1 per plant, while for N and A isolates to become the most prevalent ones in mixed infections (M isolates), aphid densities of more than 5 per plant were required and much higher transmission events (*β*_*e*_ = 0.06). This is an important difference respective to tomato, in which M isolates were the most prevalent in the population at aphid densities above 3 per plant for *β*_*e*_ = 0.03 (Fig. 1A). These results reflect that melon is a poorer host for CMV multiplication and transmission than tomato. Another important difference between hosts is that average virulence of the virus population steadily increased with *i* and *β*_*e*_ in melon, while it showed a relative minimum in tomato for low *β*_*e*_ values and moderate aphid densities (Fig. 1A). Variation of the initial conditions did not change the outcome of the simulations.

**Table 2:** Basic reproductive value, *R*_0_, for Y, A and N isolates and different aphid vector densities*

Genotype	*i* = 1	*i* = 5	*i* = 10
Tomato			
Y	9.6658	17.8565	18.2717
A	5.9452	16.7959	19.8660
N	1.8784	6.2921	8.3515
Melon			
Y	1.1375	2.9982	3.4120
A	0.4251	1.2904	1.5946
N	0.3191	1.1058	1.5129

* *R*_0_ was calculated for different aphid densities and for *β*_*e*_ = 0.05. For A and N isolates, values are mean for at least five isolates. *i* = Number of aphids per plant.

### Evolution of CMV virulence in two hosts growing synchronically

In the analysis of CMV virulence evolution in a two-host system, we considered first the situation in which both hosts, that is, tomato (Host 1) and melon (Host 2), grow during the same period within the year. This is a realistic condition that could represent the case of synchronous crops, but also the case of weeds (i.e. Host 2) growing within the crop (i.e. Host 1). Because either host may be a source of inoculum for the other, we considered the same or different rates of transmission events (*β*_*e*_) within and between hosts. Equal values of *β*_*e*_ within and between hosts imply a close spatial proximity between hosts and no vector preference for one host, while different *β*_*e*_ values within and between hosts might be due to spatial partition of host distribution and/or vector host preference. Both are realistic assumptions.

We considered first equal rates of transmission events within and between hosts. The model was simulated for *β*_*e*_ values between 0.01 and 0.15 and for *i* values of 0.5–30 aphids per plant. Initial conditions were *S*_2_ = 0.77, *Y*_2_ *= N*_2_ *= A*_2_ = 0.01, *M*_2_ = 0 plants/m^2^ and *S*_1_ = 4.0, *Y*_1_ *= N*_1_ *= A*_1_ = *M*_1_ = 0 plants/m^2^, that is, Host 2 was the inoculum source for Host 1. As before, changes in the genetic composition and in average virulence of the virus population at equilibrium could be determined (Fig. 1B). Under the above assumptions, inoculum flows between hosts resulted in very similar dynamics of Y, N, A and M isolates for both of them. For *β*_*e*_ < 0.01 and *i* < 1, Y isolates were the most prevalent in both populations whenever there was infection, as *i* increased from 1 to 3, A isolates became more prevalent than Y, and for *i* ≥ 2 and *β*_*e*_ ≥ 0.02, M isolates were the most prevalent in the populations. A summary of these results is shown in Fig. 1B. Note that virulence evolution showed different trajectories in each host, with average virulence having a relative minimum in tomato, and a relative maximum in melon, pending on *i* and *β*_*e*_ values. The comparison with Fig. 1A clearly shows the effect of inoculum flows from Host 1 to Host 2 in the dynamics of infection in Host 2. Varying the initial conditions or making Host 1, the initial inoculum source for Host 2 did not change these results (not shown). For all initial conditions, equilibrium densities of *S, Y, A, N* and *M* plants were reached faster the higher the transmission rates (not shown).

**Figure 2:**
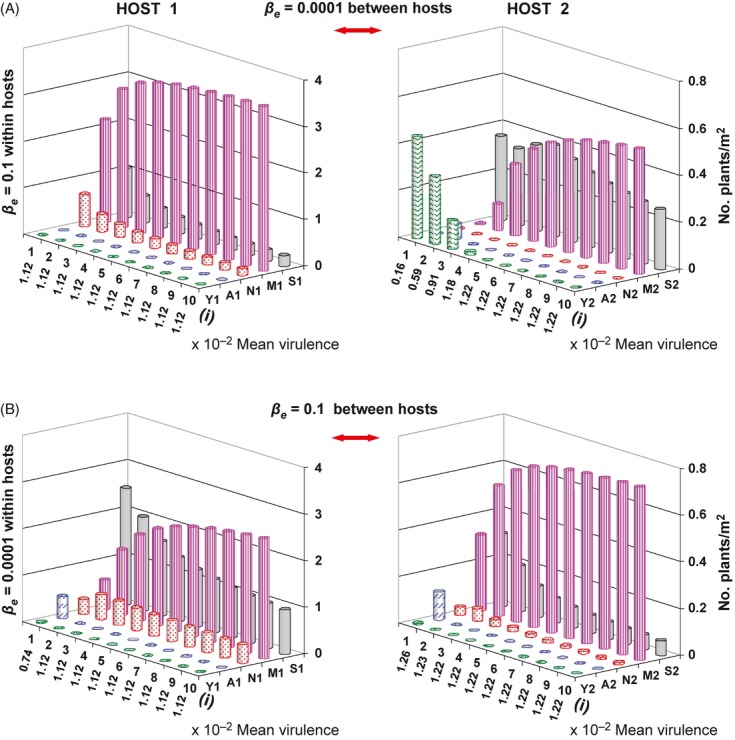
Equilibrium density of susceptible noninfected plants, S, and of plants infected by CMV isolates Y, A, N or mixed infected by CMV isolates A+N (M) according to a co-infection model for two hosts, when within-host and between-host rates of transmission events (*β*_*e*_) differ. Presented results are for within-host *β*_*e*_ = 0.1 and between-host *β*_*e*_ = 0.0001 (A) or when within-host *β*_*e*_ = 0.0001 and between-host *β*_*e*_ = 0.1 (B). Number of aphids per plant, *i*, varied from 1 to 10. Initial conditions were as follows: *S*_1_ = 3.97, *Y*_1_ = *A*_1_ = *N*_1_ = 0.01, *M*_1_ = 0 and *S*_2_ = 0.8, *Y*_2_ = *A*_2_ = *N*_2_ = *M*_2_ = 0 plants*/*m^2^. Bars represent plant density for different *i* values for noninfected plants (*S*_1_; *S*_2_ 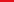 ) or for plants infected by CMV isolates Y_1_, Y_2_ ( 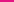 ), A_1_, A_2_ ( 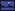 ), N_1_, N_2_ ( 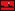 ) or mixed infected with A+N isolates (M_1_, M_2_ 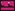).

In nature, it might be more frequent that rates of transmission events are different within hosts than between hosts and, specifically, that they are higher within than between hosts. However, simulations were done exploring all possibilities, so that *β*_*e*_ varied within and between hosts in the range 0.0001–0.1; and for the different *β*_*e*_ values, *i* varied between 0.5 and 10 aphids per plant. Results on the predicted densities of *S, Y, A, N* and *M* plants, and average virulences, are summarized in Fig. 2 for the extreme values of within- and between-host rates of transmission events and for initial conditions in which Host 1 was the infected host (*S*_1_ = 3.97, *Y*_1_ *= N*_1_ *= A*_1_ = 0.1, *M*_1_ = 0 plants/m^2^; *S*_2_ = 0.8, *Y*_2_ *= N*_2_ *= A*_2_ = *M*_2_ = 0 plants/m^2^). The reduction in the between-host values of *β*_*e*_ had a higher impact on Host 2 than on Host 1: when between host *β*_*e*_ = 0.0001, it was required that *i* ≥ 2 for A, N or M CMV isolates to have any frequency, and *i* ≥ 8 for mixed infections of A and N (M isolates) to have a frequency ≥50% in Host 2 (Fig. 2A). On the other hand, reducing within-host *β*_*e*_ had a bigger effect on Host 1, as it could reduce the frequency of infected plants (all types) below 25% (Fig. 2B). Increasing between-host *β*_*e*_ resulted in higher frequency of *M* plants in Host 2 (Fig. 2B). Note that variation in within- and between-host *β*_*e*_ values had a limited effect on the average virulence of the virus population in Host 1, in spite of its dramatic effects on infection frequency, while the reduction in between-host *β*_*e*_ values resulted in a reduction in both infection frequency and average virulence in Host 2, particularly noticeable at *i* < 5 (Fig. 2). Thus, virulence evolved to different values in each host according to transmission rates and vector densities, bifurcating at the lower vector densities.

**Figure 3:**
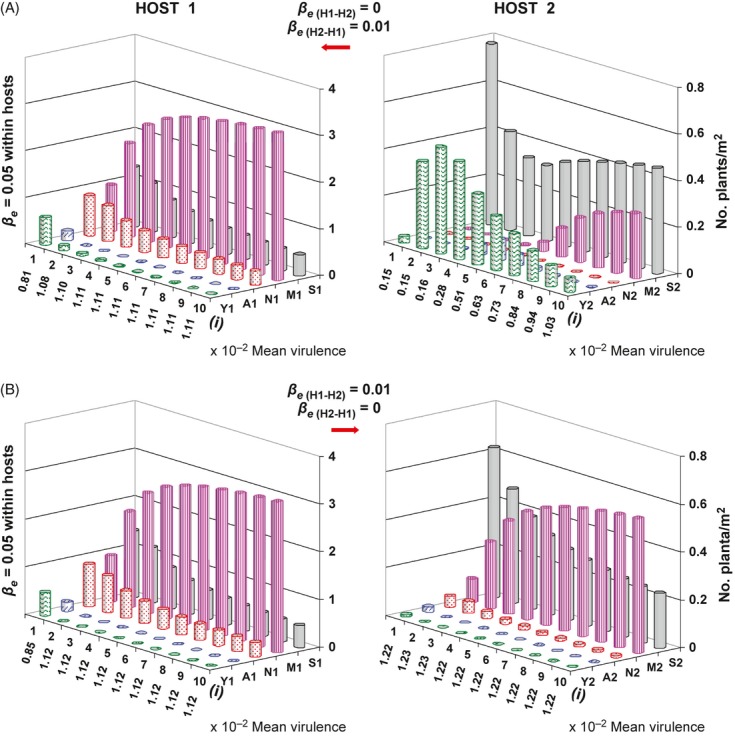
Equilibrium density of susceptible noninfected plants, *S*, and of plants infected by CMV isolates Y, A, N or mixed infected by CMV isolates A+N (M) according to a co-infection model for two hosts when within-host and between-host rates of transmission events (*β*_*e*_) differ. Presented results are for within-host *β*_*e*_ = 0.05 and transmission events from Host 1 to Host 2 *β*_*e(H*1*–H*2*)*_ = 0 and from Host 2 to Host 1 *β*_*e(H*2*–H*1*)*_ = 0.01 (A) or when within-host *β*_*e*_ = 0.05 and transmission events from Host 1 to Host 2 *β*_*e(H*1*–H*2*)*_ = 0.01 and from Host 2 to Host 1 *β*_*e(H*2*–H*1*)*_ = 0 (B). Number of aphids per plant, *i*, varied from 1 to 10. Initial conditions were as follows: *S*_1_ = 3.97, *Y*_1_ = *A*_1_ = *N*_1_ = 0.01, *M*_1_ = 0 and *S*_2_ = 0.8, *Y*_2_ = *A*_2_ = *N*_2_ = *M*_2_ = 0 plants/m^2^. Bars represent plant density for different *i* values for noninfected plants (*S*_1_; *S*_2_ 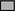 ) or for plants infected by CMV isolates Y_1_, Y_2_ ( 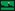 ), A_1_, A_2_ ( 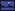 ), N_1_, N_2_ ( 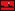 ) or mixed infected with A+N isolates (M_1_, M_2_ 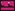 ).

Last, we considered the situation in which *β*_*e*_ values differing within and between hosts also represent asymmetric inoculum flows between both hosts. Figure 3 summarizes the results for the extreme situation in which inoculum flows only occurred from Host 2 to Host 1 (Fig. 3A) or *vice versa* (Fig. 3B). If inoculum flow occurred from Host 2 to Host 1, it sufficed that *i* ≥ 1 for Host 1 to become infected with genotypes Y and N and for mixed infections of N+A (*M* plants) reaching a high frequency. At these aphid densities, though, Host 2 was only infected by Y isolates, and much higher aphid densities (*i* ≥ 7) were required for N isolates to occur in mixed infection with A isolates and for *M* plants to be the most frequent infected plants; for all *i* values, *S* plants were the most prevalent (Fig. 3A). If transmission occurred from Host 1 to Host 2, the dynamics of infection in Host 1 was little affected, while in Host 2, high frequency of mixed infections occurred at much lower aphid densities (*i* ≥ 2, Fig. 3B). Thus, both hosts showed different sensitivity to variation of inoculum flow from the other one: in Host 1, the dynamics of infection was quite independent of inoculum flows from Host 2, while in Host 2, it was highly dependent on transmission from Host 1. In other words, in Host 1 within-host transmission is more relevant than between-host transmission, while for Host 2, transmission from Host 1 is more relevant than within-host transmission. This conclusion was also reached in simulations in which within-host transmission was not allowed (not shown). However, Host 2 was not irrelevant for infection dynamics in Host 1, as it could be a highly efficient inoculum source for Host 1. Note that when direction of inoculums transmission between hosts changed, the average virulence did not vary in Host 1 in parallel with infection frequency, while it dramatically changed in Host 2 (compare virulence in both hosts for *i* ≤ 7, Fig. 3A,B). Thus, bifurcation occurred between hosts pending on between-host transmission values.

### Evolution of CMV virulence in two hosts that rotate in time

In agroecosystems, it frequently occurs that different hosts of the same pathogen (either crops or weeds) have different growing cycles along the year (i.e. rotate temporally) so that they are alternatively inoculum reservoirs for the other hosts. This situation was simulated by making Host 1 and Host 2 rotate in time, with different time overlaps between their biological cycles and, again, considering the same or different *β*_*e*_ values within and between hosts. The results largely agree with the conclusion from the previous section in that the dynamics of infection in Host 1 was largely independent of transmission from Host 2, once infection had started, while the dynamics of infection in Host 2 was largely determined by the continuous transmission from Host 1. For simplicity, we shall present only the simulations in which the rate of transmission events, *β*_*e*_, was different within and between hosts.

**Figure 4:**
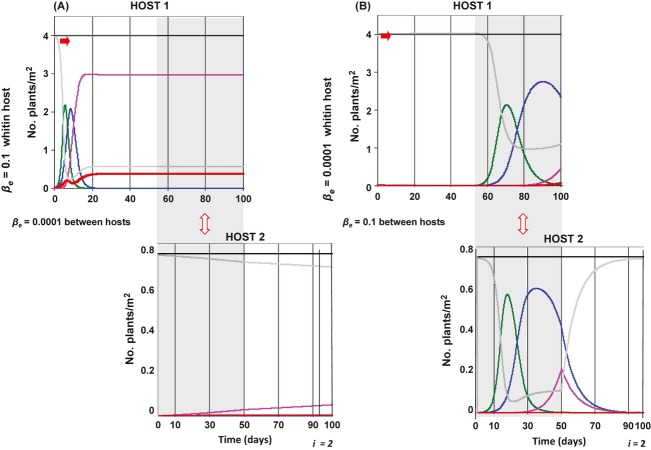
Dynamics of the populations of susceptible noninfected plants, *S*, and of plants infected by CMV isolates Y, A, N or mixed infected by CMV isolates A+N (M) according to a co-infection model for two hosts that rotate in time. Presented results are for a temporal overlap of hosts equivalent to half their life cycle (i.e. for 50 days) and for within-host *β*_*e*_ = 0.1 and between-host *β*_*e*_ = 0.0001 (A) or within-host *β*_*e*_ = 0.0001 and between-host *β*_*e*_ = 0.1 (B), for *i* = 0.5 and *i* = 2. Initial conditions were as follows: *S*_1_ = 3.97, *Y*_1_ = *A*_1_ = *N*_1_ = 0.01, *M*_1_ = 0 and *S*_2_ = 0.8, *Y*_2_ = *A*_2_ = *N*_2_ = *M*_2_ = 0 plants/m^2^. Curves represent the variation in time of the density of noninfected plants (S 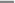 ) or plants infected by Y isolates (Y 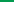 ), by A isolates (A 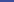 ), by N isolates (N 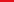 ) or by A+N isolates (M 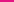 ). The shadow indicates the overlapping of the life cycles of Host 1 and Host 2, and the arrow indicates the host that initiates the rotation.

Simulations were done for within- and between-host *β*_*e*_ values varying from 0.0001 to 0.1, for each *β*_*e*_ value *i* varying between 0.5 and 30 aphids per plant, and for conditions in which Host 1 initiates the rotation and is thus the inoculum source for Host 2, and *vice versa*. Figure 4 presents the results for the extreme *β*_*e*_ values and for initial conditions: *S*_1_ = 3.97, *Y*_1_ *= N*_1_ *= A*_1_ = 0.1, *M*_1_ = 0 plants/m^2^; *S*_2_ = 0.8, *Y*_2_ *= N*_2_ *= A*_2_ = *M*_2_ = 0 plants/m^2^. When the rate of transmission events between hosts was very low, *β*_*e*_ = 0.0001, the prevalence of infection in Host 2 was below 5%. The virus genotypes that were transmitted between hosts depended on their prevalence at the end of the overlapping period between hosts, for instance in Fig. 4A, N+A in mixed infection were the most prevalent in Host 1 at the end of its growth period and were those transmitted to Host 2. However, N or A genotypes cannot be maintained in Host 2 for aphid densities of i ≤ 2. Conversely, if intrahost *β*_*e*_ values are very low (Fig. 4B), the prevalence of infected plants in Host 1 will be very low until the temporal overlap with Host 2. Thus, in this extreme situation, the dynamics of infection in Host 1 depends on transmission from Host 2.

**Figure 5:**
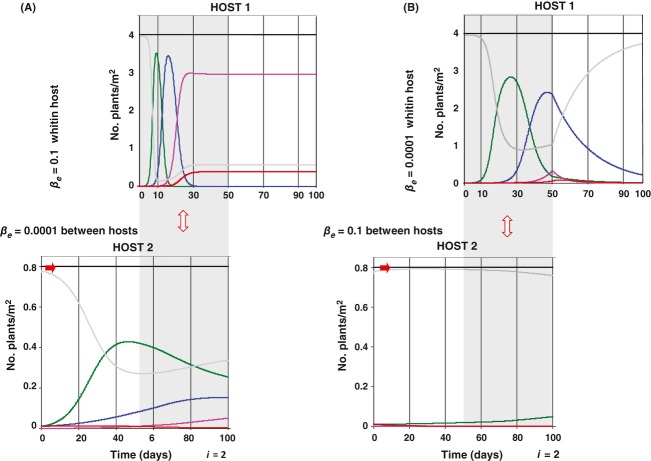
Dynamics of the populations of susceptible noninfected plants, *S*, and of plants infected by CMV isolates Y, A, N or mixed infected by CMV isolates A+N (M) according to a co-infection model for two hosts that rotate in time. Presented results are for a temporal overlap of hosts equivalent to half their life cycle (i.e. for 50 days) and for within-host *β*_*e*_ = 0.1 and between-host *β*_*e*_ = 0.0001 (A) or within-host *β*_*e*_ = 0.0001 and between-host *β*_*e*_ = 0.1 (B), for *i* = 0.5 and *i* = 2. Initial conditions were as follows: *S*_1_ = 4, *Y*_1_ = *A*_1_ = *N*_1_ = *M*_1_ = 0 and *S*_2_ = 0.77, *Y*_2_ = *A*_2_ = *N*_2_ = 0.01, *M*_2_ = 0 plants/m^2^. Curves represent the variation in time of the density of noninfected plants (S 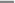 ) or plants infected by Y isolates (Y 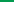 ), by A isolates (A 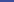 ), by N isolates (N 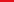 ) or by A+N isolates (M 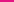 ). The shadow indicates the overlapping of the life cycles of Host 2 and Host 1, and the arrow indicates the host that initiates the rotation.

Figure 5 summarizes the results of simulations in which the rotation was initiated by Host 2 (initial conditions: *S*_2_ = 0.77, *Y*_2_ *= N*_2_ *= A*_2_ = 0.1, *M*_2_ = 0 plants/m^2^; *S*_1_ = 4, *Y*_1_ *= N*_1_ *= A*_1_ = *M*_1_ = 0 plants/m^2^). Figure 5A shows that if within-host *β*_*e*_ values are high, prevalence of Y, A or M types in Host 1 can be high, even at low between-host *β*_*e*_ values. When within-host *β*_*e*_ values were low (Fig. 5B), prevalence of infection in Host 2 was always low and in Host 1 only increased during the overlapping period, thus depending on inoculum flows from Host 2.

Modifying the overlapping period between hosts did not affect the above conclusions (not shown). It was shown than an overlap period of 10 days was sufficient for infection of the noninfected host. Under this low overlapping period, conditions for infection of Host 2 from Host 1 were between-host *β*_*e*_ ≥ 0.01, *i* ≥ 1, and condition for infection of Host 1 from Host 2 were between-host *β*_*e*_ ≥ 0.02, *i* ≥ 5, again underlining that Host 2 is a poorer host and, hence, a poorer inoculum source than Host 1.

## Discussion

In this work, we analyse the conditions that may determine the invasion of the population of a generalist plant virus, CMV, by genotypes highly pathogenic for a focal host, resulting in the emergence of a new disease syndrome. For this, we consider the evolution of CMV virulence in the focal host, tomato (Host 1), and in other hosts, exemplified by melon (Host 2). The within-host multiplication and the virulence of the different CMV genotypes differ in both hosts (Escriu et al. 2003; Betancourt et al. 2011). For this analysis, we have used a model that allows for co-infection of a single host by different genotypes which compete, the outcome of the competition affecting the transmission rates to new hosts. This model had been developed for a single-host system (Escriu et al. 2003) and was extended now to include two hosts and to allow for inoculum flows between them. Modelling virulence evolution of multihost parasites in heterogeneous host systems may be limited by a poor knowledge of the parasite's life cycle over its various hosts, what may hinder the development of models with realistic assumptions (Day 2002; Galvani 2003). The model used in this work rests on detailed epidemiological and genetic analyses of CMV and CMV-satRNA in different hosts in Spain, demonstrating that different CMV hosts may be inoculum sources with varying effectiveness for each other, that individual hosts are often infected by different CMV genotypes that compete in mixed-infected hosts and that CMV-satRNA spreads as a molecular parasite on the CMV population, converting pre-existing CMV genotypes (i.e. with no satRNA, Y isolates in this work) into new genotypes (N, A or M isolates in this work) (Jordá et al. 1992; Aranda et al. 1993; Alonso-Prados et al. 1998, 2003; Sacristán et al. 2004; Bonnet et al. 2005). These traits of CMV biology were all made explicit in the model described by eqn (2). Moreover, as our and other's work indicated that different CMV genotypes may broadly differ in phenotype according to host (García-Arenal and Palukaitis 1999; Palukaitis and García-Arenal 2003), key evolutionary parameters of within-host multiplication, within-host competition, between-host transmission and virulence were experimentally estimated (Escriu et al. 2000a,b, 2003; Betancourt et al. 2011, this work) so that model simulations could approach realistic situations.

A first conclusion of this work is that in both tomato and melon, N isolates highly virulent for tomato can invade the CMV population, but only in co-infection with A isolates, which do not differ in virulence with N isolates in melon. This conclusion agrees with field data (Alonso-Prados et al. 1998; Escriu et al. 2003). In either host, invasion of N and A isolates depended on the density of aphid vector populations, invasion being favoured by higher vector densities. The dynamics of CMV virulence in melon and tomato when considered as single-host systems followed similar patterns, with the important difference that invasion of N and A isolates in melon required much higher aphid densities than in tomato. This is the consequence of the highly relevant fact that melon-like hosts are less competent hosts for the multiplication and transmission of CMV and, specifically, of satRNAs than tomato-like hosts (Escriu et al. 2000a; Betancourt et al. 2011). On both hosts, the most virulent virus genotypes are favoured when transmission is less limiting, that is, at higher vector densities. Note, however, that at no vector density, between-host trade-offs occur in the analysed two-host system, as both within-host multiplication and the probability of transmission per contact event (*β*_*p*_) ranged similarly for the three CMV genotypes in both hosts (Y>A>N, Escriu et al. 2000a; Betancourt et al. 2011; Table 1), in spite that the transmission rate of each virus genotype varied with vector density in a host-specific way. Note also that between-host trade-offs, which have been identified as central determinants of virulence evolution in heterogeneous host systems, have been estimated seldom (Ganusov et al. 2002; Osnas and Dobson 2011), and it is uncertain how generally they occur in multihost parasites.

The second key factor for virulence evolution in heterogeneous host systems, effectiveness of between-host transmission (Gandon 2004; Osnas and Dobson 2011; Williams 2012), was made to vary in simulations of the model within ranges compatible with apparent infection rates of CMV disease progress curves (Alonso-Prados et al. 2003). When inoculum flows were allowed between Host 1 and Host 2, the model predicted the evolution of the CMV population to high virulence levels in both hosts, again as mixed N+A infections, and depend on the rate of between-host contacts (*β*_*e*_), thus again on aphid population densities. Interestingly, in both hosts, the average virulence of mixed infections was that of the more virulent genotype (N for Host 1, N and A for Host 2), a condition that according to some analyses should prevent genotype co-existence in mixed infections (Alizon 2008). Within most of the range of simulated between-host and within-host rates of transmission events, the dynamics of CMV evolution in the species that is a more competent host for CMV and, particularly, for CMV-satRNA, that is, Host 1, was quite independent of transmission from the other host, while for the less competent Host 2, dependency on transmission from Host 1 was central. Thus, between-host transmission had the effect of reducing the vector density required for the invasion of Host 2 by highly virulent genotypes, due to flows from Host 1. Under these conditions, the dynamics of genotype CMV evolution in both hosts was similar: at high vector density, N and A genotypes invaded the CMV population in mixed infection, and at low vector densities, Y genotypes predominated, again in agreement with field observations (Alonso-Prados et al. 1998; Escriu et al. 2003). However, because the three CMV genotypes do not range similarly for virulence in Host 1 and Host 2 (see Table 1), virulence evolution differs in both hosts: at high vector density, the highly virulent genotypes N (for Host 1) or N and A (for Host 2) prevail, while at low vector densities, virulence drops to intermediate (Host 1) or to the lowest (Host 2) levels. Thus, the differential effect of vector densities on virulence evolution over hosts results in a situation that is more complex than that predicted in theoretical analyses that give as an outcome of host heterogeneity either lower virulence over hosts (Ebert and Hamilton 1996; Regoes et al. 2000; Gandon et al. 2002; Dobson 2004; Gandon 2004; Williams 2012) or different evolutionary pathways in each host (Dobson 2004; Gandon 2004). Our results show that evolutionary pathways differ between hosts, that is, virulence bifurcates, when aphid densities are lower, at odds with other predictions (Gandon 2004). This difference between our results and theoretical predictions may derive from the fact that in our system, the genotypes with different virulence are not differentially adapted to each host, that is, there is not a trade-off across hosts, as pointed out above. Our results agree better with the predictions of Ganusov et al. (2002), which do not assume such a trade-off. More generally, in our system, there is not a trade-off between virulence and transmission (see Table 1) as assumed by most theory. An interesting outcome of our analyses is that the effects of vector density on average virulence at equilibrium may differ broadly from its effects on infection frequency, again underscoring the complexity of the system.

The results of the present work may also be relevant for the control of diseases caused by generalist plant viruses. A first conclusion derives from the asymmetrical role of low- and high-competent virus hosts as inoculum source for each other. Because the different hosts of a generalist pathogen may differ in their efficiency for within-host multiplication and between-host transmission and, hence, as inoculum sources for each other, selective elimination of specific host species may be efficient for disease control. In natural ecosystems, it has been shown that highly competent plant host species may determine the ecology of virus infection in less competent hosts, in which infection proceeds mostly by ‘spill-over’ from the most competent host (Power and Mitchell 2004; Cronin et al. 2010). If the virus is more virulent in the less competent hosts, this asymmetry may have deep consequences in ecosystem composition and dynamics (Power and Mitchell 2004; Malmstrom et al. 2005; Power et al. 2011). Differences in host competence have mostly not been considered in control strategies of viral diseases in agroecosystems: for generalist plant viruses, it has mostly been assumed that crop rotations or host elimination would be not efficient control strategies (Zitter and Simons 1980). Our results show that this may not be always the case. Thus, elimination of Host 1-like species, or avoiding their overlapping in the rotation, may result in efficient virus control in Host 2-like crops, with a cumulative effect over rotations.

The second conclusion relates to the effects of reducing the density of vector populations, an important control strategy for plant viruses, which results in a decrease in infection rates and prevalence. Theoretical analyses of the effect of virus transmission mechanisms on epidemics have shown that the reduction in vector density has a lesser effect on the prevalence of nonpersistently transmitted viruses, such as CMV, than on semi-persistent or persistent/circulative viruses (Madden et al. 2000) in agreement with empirical evidence (Perring et al. 1999). These analyses also showed the high sensitivity of nonpersistent virus epidemics to variation in the number of plants visited per day by an insect vector (Madden et al. 2000). This parameter may be approximated to the rate of transmission events, *β*_*e*_, in our model, which arrives to similar conclusions from different approaches. Hence, the interest to analyse experimentally the relationship between the rate of transmission events and vector density which to our knowledge is an unexplored subject. Our present and previous (Escriu et al. 2003) results also show that reducing the density of virus vectors may have the additional benefit of preventing the invasion of the virus population by highly virulent genotypes. Note that the effect of vector density reduction on virulence evolution would be independent on the virus transmission mechanism, as this factor was not considered in our analyses. However, the specific relationships between transmission mechanisms, vector density and virulence would require further analyses. Our results also show that the effect of vector control on virulence would be more effective in less competent hosts (Host 2), thus reducing their efficiency as reservoirs for highly competent hosts (Host 1). In addition with selective host rotation or elimination, the effects of reducing vector population density, particularly over periods of vector migration between hosts, on virus prevalence and virulence would be enhanced.

In conclusion, a model for the evolution of the virulence of a multihost plant virus, based on a detailed knowledge of the virus biology in its different hosts, was able to explain satisfactorily the emergence of highly virulent genotypes for a focal host and the long-term evolution of virulence over the different hosts. Moreover, predictions of this model under situations common in agroecosystems revealed the value of control measures that traditionally have been considered impractical. These results may help developing long-term strategies for the control of virus diseases. Interestingly, assumptions of trade-offs between different life-history traits of parasites that are central to most theory on virulence evolution in heterogeneous host systems did not hold for the system analysed here. This underscores the need to evaluate how generally do these trade-offs occur and to couple theoretical analyses with empirical and experimental knowledge on host–parasite systems.

## Data archiving statement

Most data for this study are available as Supporting Information attached to the online version of the article. Additional raw data are available at Dryad Repository: doi:10.5061/dryad.tr16k.

## Supporting Information

**Figure S1.** Flows among Host 1 populations of susceptible noninfected plants (S1) and plants infected by CMV isolates Y, A, N (Y1, A1, N1, respectively) or mixed infected with A+N isolates (M1), according to the co-infection model in eqn (2).

**Figure S2**. Relationship between the probability of transmission per transmission event, *β*_*p*,_ and the number of aphids per plant, *i*, for three CMV genotypes. Curves represent this relationship for Y isolates (), A isolates (), or N isolates ().

**Table S1.** Estimates of virulence in melon plants for Y, A and N isolates of CMV.

**Table S2.** Values of the probability of transmission of CMV isolates in melon by a single aphid.
