# Supplementary figures and images for: Virulence evolution of a generalist plant virus in a heterogeneous host system
Source: Evol Appl. 2013 May 20;6(6):875–90. doi: 10.1111/eva.12073 (PMC3779090; doi:10.1111/eva.12073)

## Slide 1
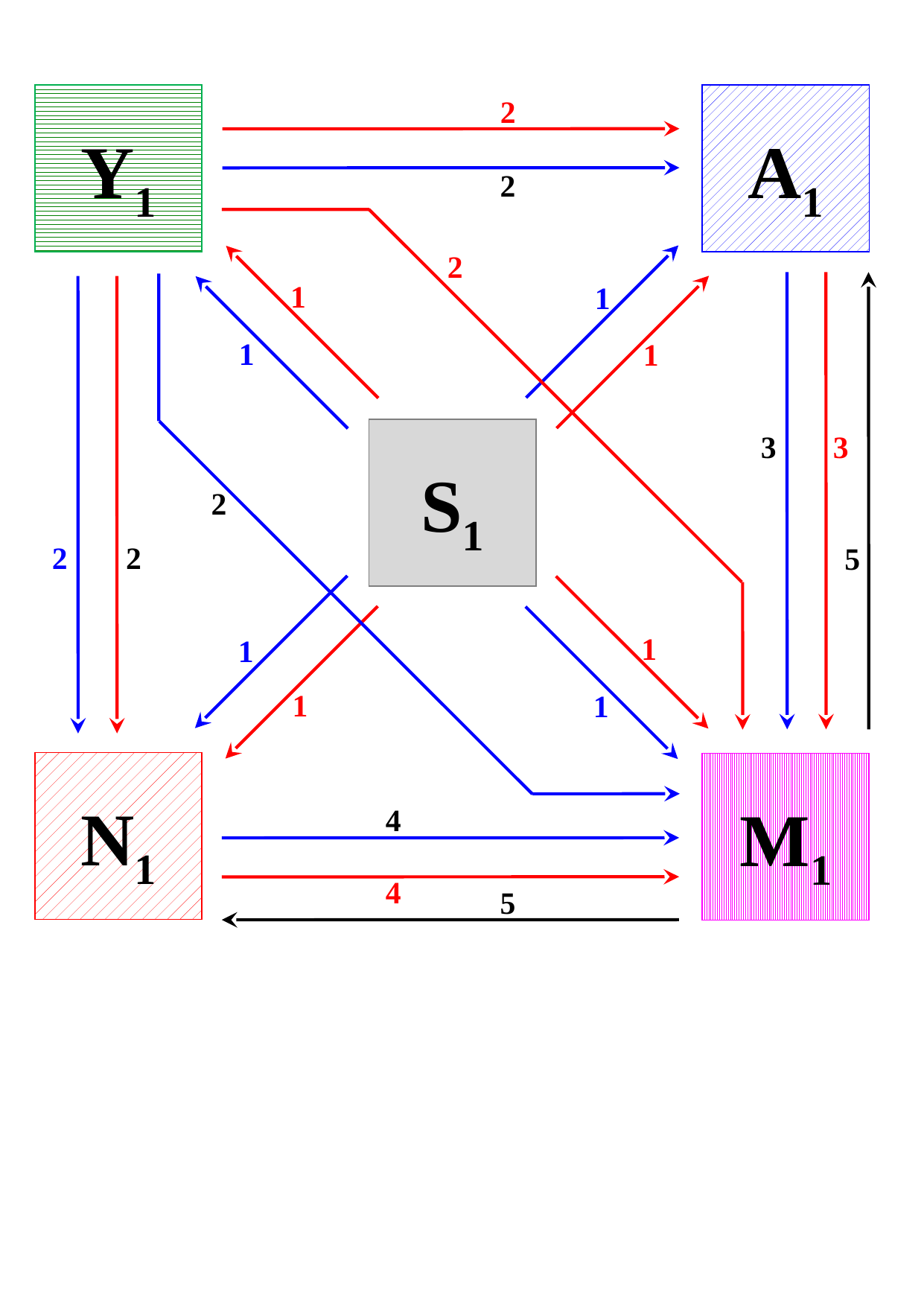

2
Y1
A1
2
2
1
1
1
1
3
3
S1
2
2
2
5
1
1
1
1
N1
M1
4
4
5

Supplement: Supplementary file 1 [file eva0006-0875-SD1.pptx]
