# Supplementary material for: Virulence evolution of a generalist plant virus in a heterogeneous host system
Source: Evol Appl. 2013 May 20;6(6):875–90. doi: 10.1111/eva.12073 (PMC3779090; doi:10.1111/eva.12073)
Supplement: Supplementary file 2 [file eva0006-0875-SD2.pptx]

## Slide 1
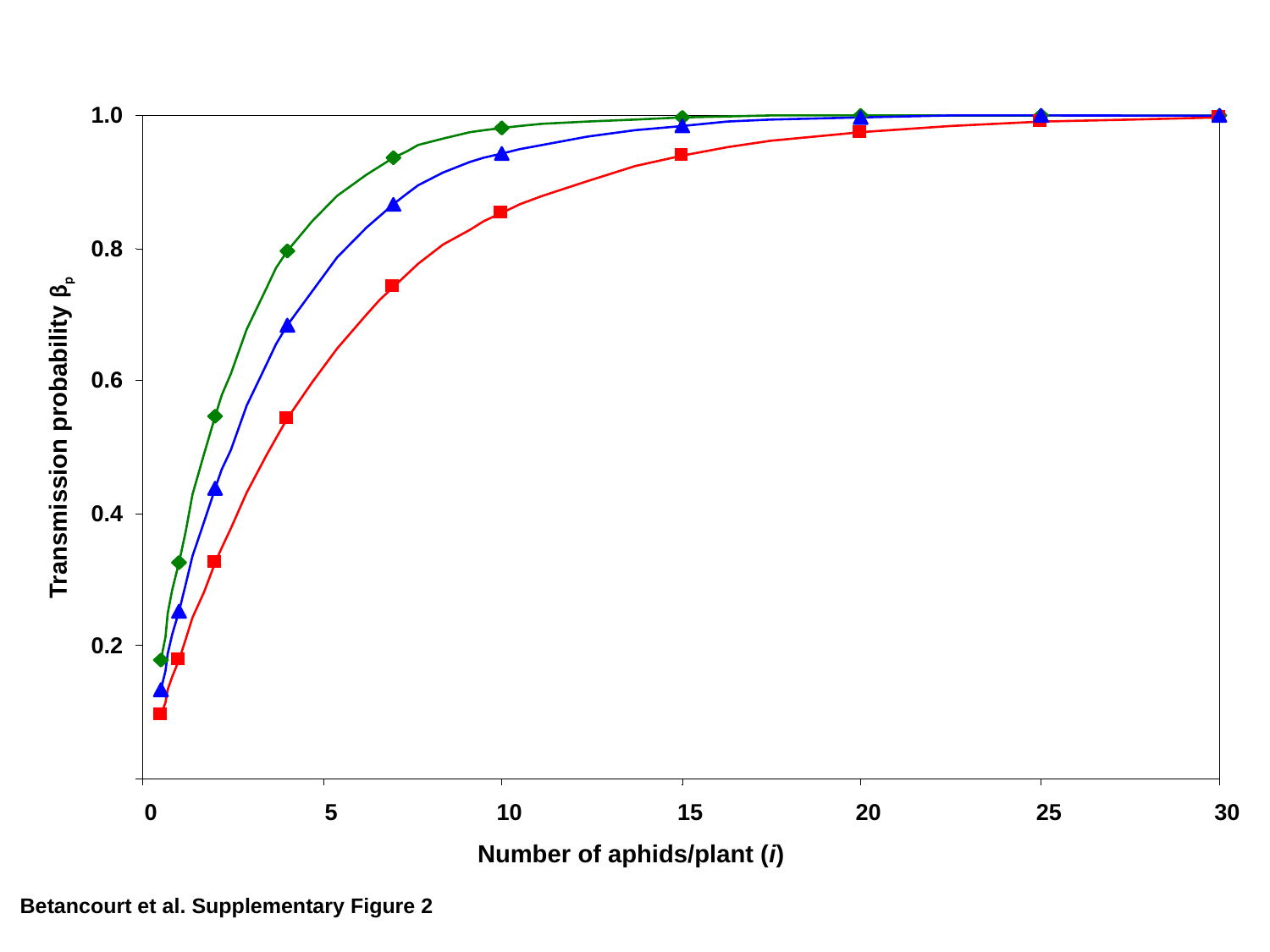

1.0
0.8
0.6
Transmission probability βp
0.4
0.2
0
5
10
15
20
25
30
Number of aphids/plant (i)
Betancourt et al. Supplementary Figure 2
