# Supplementary material for: Virulence evolution of a generalist plant virus in a heterogeneous host system
Source: Evol Appl. 2013 May 20;6(6):875–90. doi: 10.1111/eva.12073 (PMC3779090; doi:10.1111/eva.12073)
Supplement: Supplementary file 3 [file eva0006-0875-SD3.docx]

Table S1. Estimates of virulence in melon plants for Y, A and N isolates of CMV

|  |  | |
| --- | --- | --- |
| Plants | Relative duration of the plants (days, *d_j_*) | Virulence (*α_j_*) ^1^ |
| **Non infected (S)** | **1.0000** | **-** |
| **Infected by:** |  |  |
| **Genotype Y** | **0.8690** | **0.0015** |
| **Genotypes A** |  |  |
| 89/20.1 | 0.4652 | 0.0115 |
| 90/14.1 | 0.3710 | 0.0170 |
| 90/16.1 | 0.5328 | 0.0088 |
| 90/19.2 | 0.4360 | 0.0129 |
| 91/2.2 | 0.3439 | 0.0191 |
| 92/4.1 | 0.3912 | 0.0156 |
| 89/15.2^2^ | - | - |
| 89/23.1^2^ | - | - |
| 90/19.2^2^ | - |  |
| 90/14.1^2^ | - | 0- |
| **Mean + SE** | **0.4233 + 0.0283** | **0.0141 + 0.0015** |
| **Genotypes N** |  |  |
| 89/15.1 | 0.3664 | 0.0173 |
| 89/24.1 | 0.3860 | 0.0159 |
| 89/42.4 | 0.3950 | 0.0153 |
| 90/8.2 | 0.3958 | 0.0153 |
| 90/19.1 | 0.3678 | 0.0172 |
| 90/22.1 | 0.5027 | 0.0099 |
| 91/3.1 | 0.4966 | 0.0101 |
| 91/3.2 | 0.5905 | 0.0069 |
| 91/5.1 | 0.4898 | 0.0104 |
| 94/32.1 | 0.4401 | 0.0127 |
| **Mean + SE** | **0.4431 + 0.0235** | **0.0131 + 0.0011** |
| **Genotypes M (A+N)** |  |  |
| 89/15.1 | 0.3931 | 0.0154 |
| 89/24.1 | 0.4620 | 0.0116 |
| 89/42.4 | 0.4183 | 0.0139 |
| 90/8.2 | 0.4573 | 0.0119 |
| 90/19.1 | 0.4240 | 0.0136 |
| 90/22.1 | 0.4443 | 0.0125 |
| 91/3.1 | 0.4777 | 0.0109 |
| 91/3.2 | 0.4643 | 0.0115 |
| 91/5.1 | 0.4738 | 0.0111 |
| 94/32.1 | 0.5127 | 0.0095 |
| **Mean + SE** | **0.4528 + 0.0108** | **0.0122 + 0.0005** |

^1^ Data are estimates from 10 plants for single-infection treatments, and from 20 plants for mixed-infection treatments.

^2^ These satRNAs were unable to multiply in melon plants.

Table S2. Values of the probability of transmission of CMV isolates in melon by a single aphid

| **CMV genotype** | **Transmission probability^a^** |
| --- | --- |
| **Non infected (S)** | **-** |
| **Infected by:** |  |
| **Genotype Y** | **0.3270** |
| **Genotypes A** |  |
| 89/20.1 | 0.2744 |
| 90/14.1 | 0.2116 |
| 90/16.1 | 0.2326 |
| 90/19.2 | 0.2734 |
| 91/2.2 | 0.2532 |
| 92/4.1 | 0.2600 |
| 89/15.2^b^ | 0 |
| 89/23.1^b^ | 0 |
| 90/19.2^b^ | 0 |
| 90/14.1^b^ | 0 |
| **Mean + SE** | **0.2509 + 0.0100** |
| **Genotypes N** |  |
| 89/15.1 | 0.1940 |
| 89/24.1 | 0.1347 |
| 89/42.4 | 0.1471 |
| 90/8.2 | 0.1586 |
| 90/19.1 | 0.2173 |
| 90/22.1 | 0.2301 |
| 91/3.1 | 0.1827 |
| 91/3.2 | 0.1496 |
| 91/5.1 | 0.2165 |
| 94/32.1 | 0.1655 |
| **Mean + SE** | **0.1797 + 0.0106** |

^a)^ Data are estimates from 5 plants for each treatment

^b)^ These satRNAs were unable to multiply in melon plants.
